# Oxidative Stress in Brain in Amnestic Mild Cognitive Impairment

**DOI:** 10.3390/antiox12020462

**Published:** 2023-02-11

**Authors:** D. Allan Butterfield

**Affiliations:** Sanders-Brown Center on Aging, Department of Chemistry, University of Kentucky, Lexington, KY 40506, USA; dabcns@uky.edu; Tel.: +1-(859)-257-3184

**Keywords:** lipid peroxidation, Abeta and oxidative stress, neuronal loss, key metabolic pathways involved in MCI, potential therapeutic strategies for MCI

## Abstract

Amnestic mild cognitive impairment (MCI), arguably the earliest clinical stage of Alzheimer disease (AD), is characterized by normal activities of daily living but with memory issues but no dementia. Oxidative stress, with consequent damaged key proteins and lipids, are prominent even in this early state of AD. This review article outlines oxidative stress in MCI and how this can account for neuronal loss and potential therapeutic strategies to slow progression to AD.

## 1. Introduction

Alzheimer disease (AD) is the major cause of dementia worldwide. Millions of people around the globe currently have AD, and with an ever-increasing aging population globally, the number of AD patients will continue to grow at a rapid rate in the absence of effective, disease-modifying treatments.

An initial stage of many individuals who later are diagnosed with AD is amnestic mild cognitive impairment (MCI) [1]. There are two types of mild cognitive impairment, amnestic (MCI) and non-amnestic (nMCI), with the former most often associated with subsequent AD development [1]. Persons with MCI are known to have normal activities of daily living—that is, there is no dementia—but memory difficulties are the prominent clinical presentation of this disorder. The conversion rate of MCI to AD is about 15% per year of a given population of MCI patients [1].

One of the major contributors to the pathophysiology and likely progression of AD is oxidative stress in brain [2,3,4,5], defined as the oxidative damage to proteins and lipids (and other cellular components) resulting from an imbalance in favor of free radical production vs. the scavenging capabilities of antioxidants and antioxidant-related enzymes [6,7]. Oxidative damage indices, among others, include markers of protein oxidation (elevated levels of protein carbonyls (PC) and/or 3-nitrotyrosine (3-NT)) and lipid peroxidation (most often characterized by elevated levels of protein-bound 4-hydroxynonenal (HNE) and elevated levels of isoprostanes or neuroprostanes) [6,7,8,9,10,11,12,13].

This current review article focuses on oxidative stress in brains from persons with MCI, with its implications that oxidative and nitrosative damage in brain in persons with MCI, who do not demonstrate dementia, may contribute to critically important early processes that lead to the dementing disorder, AD, and, while many clinical trials of free radical scavengers have been disappointing in AD, interception of free radical processes early in the progression toward AD can slow or potentially halt progression to this devastating disorder. 

## 2. MCI Pathology and Clinical Presentation

MCI pathology was fully characterized in excellent studies by Markesbery and Morris [14,15], while clinical presentation of MCI has been reported in outstanding reports by Peterson and Jicha [16,17]. Pathologically, MCI brain is characterized similarly to AD brain, i.e., presence of senile plaques (composed mostly of aggregated fibrils of amyloid b-peptide, a 39–42 amino acid peptide that is neurotoxic, and dystrophic neurites), neurofibrillary tangles (composed of aggregates of hyperphosphorylated tau protein) and loss of synapses. However, MCI brain has these pathological hallmarks to a significantly less degree than what normally occurs in AD brain. Positron-emitting tomography images resulting from ^18^F-2-deoxyglucose showed that MCI patients have markedly decreased glucose utilization for brain function than do brains from control individuals, but significantly less than brains from persons with AD [18,19]. Clinically, amnestic mild cognitive impairment patients are capable of normal activities of daily living but do demonstrate a perceptible memory loss confirmed by a close informant [16]. 

## 3. Amyloid β-Peptide and Oxidative Stress

When small oligomers of amyloid b-peptide (Aβ42) of 42 amino acids in length are added to neurons in culture, oxidative stress results, as indexed by elevated levels of PC, protein-bound HNE, and 3-NT [20,21,22,23,24,25,26,27].

In vivo, oxidative stress, characterized by elevated PC, was observed in the roundworm, *C. elegans*, in which human Aβ42 was expressed [28,29,30]. Also, elevated oxidative stress, indexed by PC, HNE, and 3-NT, was observed in brains of mice in which human mutated amyloid precursor protein (APP) was added as a transgene or was knocked in after the mouse gene was knocked out [31,32,33,34,35,36,37,38,39,40,41,42,43,44,45,46,47,48,49,50,51,52,53,54,55,56,57,58,59,60,61,62,63,64,65,66]. Aβ42 is produced in vivo by action of both the extra-neuronal beta-amyloid cutting enzyme (BACE) and the intramembrane protease γ-secretase. Similarly, in the senescence-accelerated mouse (SAMP8), brain oxidative stress is observed, but if antisense oligonucleotides of γ-secretase or Aβ are employed, the resulting brains in SAMP8 mice no longer show elevated oxidative stress, consistent with our laboratory’s hypothesis that Aβ plays critical roles in brain oxidative stress [59,61]. Moreover, in rat brain, oxidative stress associated with Aβ42 was observed [67,68,69,70]. Brains from aged beagle dogs, which express Aβ42 of the same amino acid sequence as humans, show evidence of oxidative damage, but if the aged dogs were fed, over a 3-year period, a diet rich in antioxidants, provided an enriched environment, and exercised, these aged beagles had greatly increased cognitive performance and significantly decreased levels of oxidative damage in brains compared to similarly aged dogs fed dog chow and not exposed to behavioral enrichment [71]. Redox proteomics of brains of these canines identified less oxidatively modified proteins and associated pathways consistent with the improved cognition.

Collectively, these in vitro and in vivo studies strongly support our now well-accepted idea that Aβ42 oligomers are associated with oxidative stress [8,9,10,12,21,23,26,27,28,29,30,31,32,33,34,35,36,38,39,40,43,44,45,50,51,52,53,55,59,60,64,67].

## 4. Oxidative Stress in MCI Brains

Evidence for oxidative stress in MCI brains, indexed by PC, HNE, and 3-NT, was first reported in studies conducted at the University of Kentucky [10,72,73,74,75,76,77,78,79,80,81,82,83,84,85,86,87,88,89]. These results were confirmed by others [90,91,92,93,94,95,96,97,98,99], and are consistent with the notion that oxidative damage is an early event in the progression of AD. Our laboratory hypothesized that oligomeric Aβ42-associated oxidative damage, particularly lipid peroxidation and mitochondrial damage, underlies these results in MCI [8,9,72,73,74,75,76,77,78,79,80,81,83,84,85,86,87]. Consistent with our hypothesis, J20 mice (which have human APP_SWE,IND_ mutations) show elevated oxidative stress [50,51], but in mice with a third mutation in APP_SWE,IND_, namely substitution of the codon for Met-631 by Leu (residue 631 of APP in this mouse corresponds to the Met-35 residue of Aβ42), no elevated oxidative damage in brain was observed [32]. This last result also validates the critical role of the single Met residue of Aβ42 in the oxidative stress associated with this peptide [24,28,31,50,51,100,101]. 

Using redox proteomics on brains from well-characterized MCI individuals obtained with a post-mortem interval typically of fewer than 4 h, oxidatively modified brain proteins and the pathways in which these proteins could be placed were identified (reviewed in [87]). Table 1 lists the oxidatively modified hippocampal or inferior parietal lobule proteins from MCI brains compared to control brains identified by redox proteomics in our laboratory. When possible, functional assays were completed, and in general, oxidative damage, which changes the structure of proteins [6,102,103,104], decreases protein function.

Deposition of Aβ42 is evident in brains of those who develop AD about 20 years prior to onset of clinical presentations of this disorder [104,106,107]. Imaging-based, temporal studies of persons who were genotyped to know they carried a gene mutation for autosomal-dominant Alzheimer disease were carried out over a 22-year period prior to onset of clinical symptoms [106]: Positron-emitting tomography (PET) scanning for Aβ deposition and separately for glucose metabolism and magnetic resonance imaging analyses of hippocampal and frontal cortex thinning, an index of neuronal loss. The results indicated that Aβ deposition occurred first, followed some later time by evidence of glucose dysmetabolism, and later yet, thinning of hippocampal and frontal cortical regions of brains were observed. 

These imaging studies are highly consistent with our oxidative stress of MCI and AD brains. Specifically, Aβ deposition, the first imaging-detected abnormality in people destined to develop clinical symptoms of AD, means Aβ42 oligomers are present early in the development of inherited AD, and presumably sporadic AD as well. Significantly hydrophobic in nature, small Aβ oligomers resident in neuronal membrane lipid bilayers would lead to lipid peroxidation [9,75,108,109], resulting in HNE production and consequent oxidative modification and dysfunction of membrane lipid and cytosolic-resident proteins, such as those associated with glucose metabolism identified by redox proteomics (Table 1). Moreover, these oxidatively modified proteins can lead to neuronal death with consequent thinning of hippocampus and frontal cortex, which would cause loss of cognition. 

Redox proteomics in MCI brains (Table 1) shows oxidative modification of similarly critically important proteins involved in pathways, as observed in AD brain [8,87], again supporting the notion that oxidative damage to neurons is an early event in the pathophysiology and progression of AD. For example, glucose hypometabolism in MCI brain resulting from oxidative modification of glycolytic-related proteins (aldolase, phosphoglycerate kinase, phosphoglycerate mutase 1, a-enolase, pyruvate kinase, lactate dehydrogenase) and the mitochondrial protein complex, ATP synthase [72,76,79,81,85,105,110] (Table 1), would lead to significant diminution of ATP production. This, in turn, would cause significant loss of neuronal cell potential, which would open voltage-gated Ca^2+^ channels. Because of the 10^4^ greater Ca^2+^ concentration outside the neuron compared to the cytosolic concentration of Ca^2+^, a large influx of Ca^2+^ would overwhelm internal Ca^2+^ stores and activate both various degradative processes, such as proteases, phospholipases, and endonucleases (involved in necrotic processes), and cause mitochondrial swelling, leading to opening of the mitochondrial permeability transition pore. Cytochrome c would easily exit the mitochondria, initiating apoptotic processes. Other processes leading to neuronal death could occur, and these, coupled with necrosis and intrinsic apoptosis, would lead to a thinning of hippocampal and frontal cortical brain regions. Consistent with the discussion above about the role of Met-35 of Aβ42 in its oxidative stress-associated properties, others showed Met-35 of Aβ42 is critical to the dysregulation of intracellular Ca^2+^ homeostasis [111].

In addition to glucose dysmetabolism, loss of redox homeostasis and defense occurs in MCI brains (Table 1). Peroxiredoxin 6 (Prdx6), in contrast to the other members of the peroxiredoxin family of proteins, is the only 1-Cys enzyme that lacks a second Cys that in other members of this family of proteins serves a resolving function. Instead, Prdx6 uses glutathione coupled with glutathione-S-transferase-p as part of the catalytic cycle to restore glutathione peroxides to reduced glutathione [112,113]. HNE modification of the single catalytic Cys residue would lead to loss of Prdx6 function and contribute to oxidative-damage-induced neuronal death. Prdx6 is a pleiotropic enzyme, also demonstrating Ca^2+^-independent phospholipase A2 activity localized in lysosomes where the low pH activates the enzyme [114]. The current author speculates that oxidative modification of Prdx6 inhibits this phospholipase A2 activity and therefore inhibits full lysosomal degradation of cellular detritus that would potentially contribute to neuronal death mechanisms. Another enzyme oxidatively modified in MCI brains is carbonyl reductase, which reduces aldehydes, such as HNE, to alcohol. Consequently, oxidative modification and dysfunction of carbonyl reductase in MCI brains would contribute to the oxidative damage observed in MCI brains [8,10,72,74,76]. 

Memory and learning processes involve remodeling of synaptic membranes; small oligomers of Aβ42 are known to negatively affect these processes [115,116,117,118,119]. Interestingly, large oligomers of Aβ42 do not affect these processes [118], which is consistent with the notion that small oligomers of Aβ42 can easily insert into the lipid bilayer to participate in processes of lipid peroxidation and HNE formation, but large Aβ42 oligomers are too big to insert into neuronal synaptic membranes and therefore do not lead to lipid peroxidation or cause loss of synaptic membrane remodeling. In MCI brains, proteins involved in neurite extension (dihydropyrimidinase-related protein-2) or actin remodeling (b-actin, fascin) and in neurotransmission processes (syntaxin-binding protein-1) that are oxidatively modified, and therefore dysfunctional, likely contribute to memory and cognitive issues in amnestic mild cognitive impairment, and the current author hypothesizes that Aβ42-associated oxidative damage to these proteins plays a role in these effects. 

Proteins containing a phosphorylated Ser or Thr on the N-terminal side of a Pro residue are subject to regulation by the peptidylprolyl cis-trans isomerase (Pin1) [120,121]. Pin1 uses its WW domain to recognize this p-Ser/Thr-Pro motif on the protein to be regulated by binding to this motif. Then the active site of Pin1 rotates up via a hinge area on Pin1 to interact with the Pro residue of the protein to be regulated and converts the Pro from a cis conformation to trans conformation, or vice versa. Such an orientation shift of the Pro residue causes a major conformational change of the protein on which the p-Ser/Thr-Pro motif exists, and in that way the activity of the protein is regulated by Pin1. In MCI brain, Pin1 is oxidatively modified, as indexed by PC and HNE binding (Table 1) [72,76]. Our group showed that the activity of Pin1 is significantly depressed in AD brain, in which Pin1 also is oxidatively modified [122]. Therefore, one can confidently predict that the activity of Pin1 is similarly diminished in MCI brain, potentially by HNE binding to the Cys-containing active site. In AD brain, which has undergone lipid peroxidation for a much longer time than MCI brain, the active-site Cys residue is highly oxidized to sulfonic acid [123]. Among the key proteins in brain regulated by Pin1 are APP, from which Aβ is produced, and protein phosphatase 2A, among whose substrates is phosphorylated tau [121]. Therefore, in MCI brain, dysfunctional Pin1 no longer regulates APP and phosphorylated tau, consistent with the concept that dysfunctional Pin1 contributes to formation of the two principal hallmarks of MCI (and AD) neuropathology, senile plaques that are rich in highly aggregated Aβ42 fibrils, and hyperphosphorylated tau-containing neurofibrillary tangles.

Neuronal death following failure to remove glutamate from the synaptic cleft after glutamate neurotransmission, resulting in repeated depolarization of post-synaptic neurons, leads to neuronal death in a process known as excitotoxicity, a process first named by Onley [124]. Normally, the glutamate transporter EAAT2 (also known as Glt-1), found in astrocytes, transports Glu from the synaptic cleft to astrocytes when its role in glutamate neurotransmission is completed. In astrocytes, Glu is converted to Gln by the enzyme glutamine synthetase (GS). However, in AD brain and by Aβ42, Glt-1 is oxidatively modified [125,126] and others showed this transporter was defective in brain in this disorder [127]. This, coupled with the oxidative modification and dysfunction of GS in AD [103,128] and MCI brains [72], shows that excitotoxicity is another means of inducing neuronal death in MCI (and AD) brains. 

Redox proteomics identification in MCI brains of oxidatively modified proteins involved in signal transduction, protein folding, and protein synthesis are processes known to be altered in AD and MCI brains (Table 1), indicating that such dysfunctional pathways are early events in the progression of AD [14,129]. In the cases of HSP70 and HSP90, interactions of these proteins that are synthesized from nuclear genes but destined for mitochondria are kept in a folded state as they encounter TOM70, a mitochondrial translocase. Because HSP70 and HSP90 are oxidatively modified in MCI brain [76,81,85,86,110], their functions are likely compromised, which would have the effect of mitochondria being deprived of its full complement of proteins for effective functioning. It is well known that mitochondrial functions are deficient in AD [130,131] and MCI [86,132], and our studies showing specific mitochondrial proteins are oxidatively modified contribute to the understanding of why brain mitochondrial functions are diminished in these disorders compared to age-matched control brains.

In addition to brain, oxidative damage also occurs in mitochondria isolated from blood lymphocytes in MCI [133], which conceivably could serve as one component of a panel of biomarkers for diagnosis and treatment efficacy of this non-dementing disorder. 

## 5. Some Potential Translational Approaches to Mitigate Oxidative Damage in MCI Brains

While preclinical studies of antioxidant treatment of animal or neuronal cell culture models of AD showed efficacy against oxidative stress, clinical trials of antioxidants in AD and MCI have been for the most part quite disappointing [134,135]. There could be several reasons for these outcomes, including the nature of the antioxidant used in the clinical trials, the absorption, distribution, and elimination profiles, including ability to cross the blood-brain barrier, differences among the antioxidants employed, trials that did not have an additional means of recycling the antioxidant back to its reduced state, and the inherent redox state of individuals in the trials. However, likely one of the biggest reasons for disappointment among antioxidant-based clinical trials in MCI [136], in the opinion of the current author, is that MCI is a multifactorial disorder. So, while oxidative stress may underlie dysfunction of various, but perhaps not all relevant, pathways, the point is that there are multiple pathways involved. Antioxidants may show a preference for efficacy for one or more pathways over other pathways. Again, this author opines that multiple approaches, ranging from antioxidant pharmaceuticals, exercise, targeted nutraceuticals, insulin signaling, inhibitors of mTORC1, sufficient sleep, targeting vascular factors such as homocysteine levels, among other approaches, would need to be simultaneously employed. 

Consequently, one approach likely would not be able to cover all the factors involved in such a complex disorder as MCI. There are far more clinical trials of antioxidants and other approaches reported for AD compared for MCI (a PubMed search shows 733 papers for antioxidant clinical trials in AD; 276 for MCI; and 133 for both). Consequently, brief vignettes of the results of two selected antioxidant-based clinical trials in MCI are presented in the following: 

A three-year, double-blind clinical trial involving 769 MCI participants treated daily with placebo, 2000 IU of vitamin E, or 10 mg of donepezil was conducted [136]. Vitamin E demonstrated no improvement in MCI patients; in contrast, donepezil treatment, compared to placebo, led to a lower rate of progression to AD early in the treatment period. 

A combination approach of N-acetyl cysteine, α- tocopherol, acetyl-L-carnitine, vitamin B12, folic acid and S-adenosylmethionine was used for one year in a small trial of 10 MCI patients. No statistical differences between nutraceutical and placebo group were observed [137], likely due to the small number of MCI patients involved. A later clinical trial of the same combinational approach employing a much larger cohort of MCI patients showed no change or improved cognitive results [138,139].

## 6. Conclusions about and Future Prospects for Studies of Oxidative Damage in MCI Brains 

It is clear from studies from our laboratory and others that oxidative damage in MCI brain evidenced by protein oxidation (indexed by elevated levels of protein carbonyls and protein-resident 3-nitrotyrosine) and lipid peroxidation (indexed by increased levels of protein-bound HNE) are early events in the progression of AD that occur well before the onset of dementia. Therefore, studies to understand more the relationships among oxidative damage in MCI brain with the pathophysiology and progression of AD are important areas of study.

Recently, the pharmaceutical agent lecanemab, which preferentially targets protofibrils of Aβ, reportedly led to a significant and modest decreased rate of cognitive decline in AD patients, arguably the first disease-modifying drug for AD [140]. In this author’s opinion, this finding supports the notion that amyloid beta-peptide small aggregates play important roles in the cognitive loss of AD. It would be interesting to know if preclinical studies of lecanemab-treated AD mouse models would demonstrate significantly improved cognitive performance accompanied by decreased oxidative damage in brain. Moreover, even more profound results conceivably would be obtained with drugs that preferentially inhibit the formation of oligomers of Aβ42, which are precursors of protofibrils. Moreover, since lecanemab also is associated with detrimental issues of bleeding in the parenchyma, a different drug targeted at small Aβ42 oligomers, which, as noted above, are reportedly detrimental to synaptic long-term potentiation and depression (normally associated with learning and memory) [115,116,117,118] and involved in lipid peroxidation and neuronal death [3,8,8,9,10,12,37,74], plausibly would be more beneficial in slowing cognitive loss than lecanemab and without the associated side-effects of lecanemab. 

Given the aging of the large population of baby boomers in the United States, in the absence of profoundly disease-modifying agents, AD will become a financially intolerable burden on the people and government of this country. Moreover, given the extensive neuronal loss in AD patients, effective treatment necessarily will need to commence at least in the MCI stage of AD. In addition, given the complexity of the molecular bases of MCI and AD, in this author’s opinion, a multi-pronged attack of affected pathways and therapeutic approaches will be needed (i.e., see the elegant studies of Nixon and colleagues on endosomal-lysosomal alterations, including Aβ deposition, related to neuronal death [141]). 

Targeting simultaneously the oxidative damage to numerous pathways in MCI brain, as outlined in this review article, coupled with behavioral modifications such as high-antioxidant diets, exercise, brain stimulation by developing new skills, and routinely getting sufficient sleep, will, in this author’s opinion, be necessary to lead to a significant retardation of the rate of cognitive decline. All this would require definitive means of predicting with high accuracy and precision who will develop AD based on relatively easily obtained biomarkers, of which a growing list of candidates is gaining attention [142,143]. One such biomarker is elevated levels of homocysteine, which can lead to oxidative stress, cleavage of DNA, and apoptosis [144,145,146,147]. Finally, given that the presence of AD pathology develops nearly 20 years prior to development of AD symptoms, such screening, in this author’s opinion, needs to begin perhaps at the age that mammograms and colonoscopies are recommended to begin. Specifically, for colonoscopies, the American Cancer Society recommends starting regular screening at age 45, and starting screening with mammograms at age 40 (optional) or 45 (should be screened). Discussions among scientists, physicians, and ethicists to develop plans to address this oncoming surge in AD, in the absence of highly efficient means of decreasing development of AD, should be taking place now. With advances in therapy, identifying who is likely to develop symptoms of AD two decades later would provide neurologists with knowledge to begin imaging screening for AD pathology, along with interventions to significantly increase the age at which AD symptoms might develop, thereby increasing the quality of life in aging individuals.

**Figure 1:**
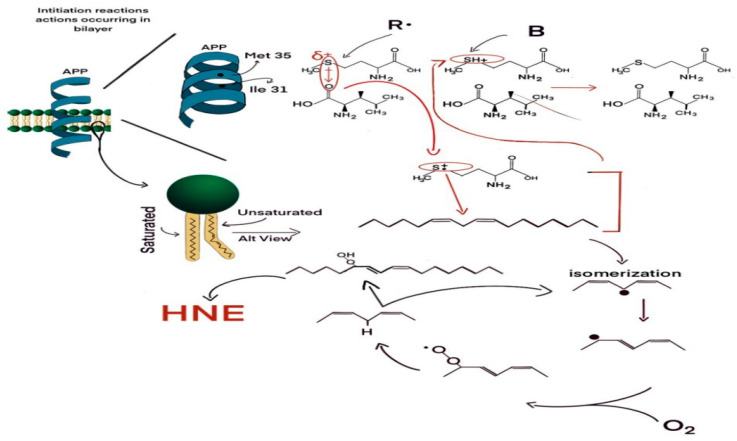
Proposed mechanism for formation of the highly reactive and neurotoxic product of lipid peroxidation, 4-hydroxynonenal (HNE).

Figure 1 displays a closer look inside the neuronal plasma membrane lipid bilayer in which is inserted small oligomers of amyloid b-peptide, with each monomer of 42 amino acids in length. Like other lipid-soluble proteins, small Aβ42 oligomers (for example, dimers, tetramers) adopt an alpha-helical structure for those amino acids resident within the lipid bilayer. As such, each amino acid interacts with the fourth amino acid away. In this case, the carbonyl moiety of Ile-31 is within a van der Waals distance (3.7 Angstroms) of the sulfur atom of Met-32 of Aβ42. The oxygen atom, being more electronegative than the sulfur atom, draws electron density slightly away from the sulfur nucleus, making a lone pair of S electrons vulnerable to a one-electron oxidation (i.e., loss of the electron). A radical R. in the lipid bilayer attacks the S lone pair to satisfy its valence shell need and produces a S^●+^ sulfuranyl free radical. This, in turn, attacks a labile allylic H-atom attached to a C atom one carbon removed from a double bond in the fatty acid chain of the phospholipid (for example, arachidonic acid, which is rich in neuronal membranes), forming the SH^+^ moiety on the Met and a carbon-centered free radical on the fatty acid chain of the phospholipid or sphingolipid, C. This free electron moves to a different carbon atom upon which molecular oxygen (which has two separate unpaired electrons and, due to its zero dipole moment, is highly soluble in the hydrophobic environment of a lipid bilayer) reacts in a radical-radical recombination reaction (often the fastest-known reactions) to form the lipid peroxyl free radical, LOO. This free radical (the second unpaired electron from molecular oxygen) can then attack a different labile allylic H-atom on a fatty acid chain, forming the lipid hydroperoxide, LOOH, and another carbon-centered free radical, C^●^. The LOOH lipid hydroperoxide is the source of the HNE, formed by a series of reactions too complex to show here. Note that the production of a new carbon-centered free radical, which then triggers the same reactions noted above to produce more HNE, is a chain reaction. That means two critical outcomes emerge: (a) This reaction will continue as long as there is molecular oxygen and allylic H-atoms present; and (b) a small amount of initial formation of the S^●+^ free radical on Aβ42 is sufficient to produce a huge amount of neurotoxic HNE, i.e., the level of HNE formed is greatly amplified by the chain reactions of lipid peroxidation. Looking at the SH+ moiety formed, this is an acid in the sense of an atom with a H on it. This acid has a pKa of −5, meaning that any weak base B, for example water, can immediately remove the H+ from the S-atom of Met-35, reforming the original methionine residue of Aβ42. That is, this is a catalytic reaction as well, in which the secondary structure of the Aβ42 oligomers in the bilayer contributes to its own reactivity. Moreover, an alpha-helix has its own dipole moment, which could stabilize the sulfuranyl free radical, S^●+^, long enough to allow attack of this free radical on the allylic H-atom to initiate the lipid peroxidation process. The alpha-helical dipole moment is dependent on the length of the helix, which conceivably could contribute to the known greater neurotoxicity of Aβ42 compared to Aβ40. What is the nature of the radical, R^●^, in the lipid bilayer? Two considerations are relevant: (a) The radical must reside within the bilayer, since a radical reactive enough to pull an electron off the S-atom of Met, as a consequence of the alpha-helix secondary structure of the lipid bilayer-resident portion of the peptide, would be too reactive to survive the transit from outside the membrane to inside the membrane. Rather, the radical needs to be within a van der Waals distance from the S-atom of Met; (b) the nature of R. is unknown, but molecular oxygen could be a likely candidate. Molecular oxygen, which after abstracting an electron from the S-atom of Met would become the superoxide free radical anion, O_2_^●−^, can immediately form hydrogen peroxide, H_2_O_2_, which, in the presence of the reduced forms of iron or copper (Fe^2+^, Cu^+^), respectively, can form the powerful and dangerous hydroxyl free radical (HO). As noted above in this figure legend, molecular oxygen is highly soluble in the hydrophobic environment of the lipid bilayer. See the text for more explanation of how significant amplification of highly neurotoxic HNE can be formed by a relatively small amount of radical formation on the Met atom of Aβ42.

## Figures and Tables

**Table 1:** Oxidatively modified proteins and their corresponding functional grouping from different brain regions in MCI compared to control brains.

Function	Protein	Brain Region: Hippocampus (H); Inferior Parietal Lobule (I)	Elevated Oxidative Stress Index: Protein Carbonyls (PC); Protein-Bound HNE (HNE); Protein-Resident 3-NitroTyrosine (3-NT)	Reference
Glucose Metabolism	Aldolase	I	3-NT	[85]
	Phosphoglycerate kinase	H	HNE	[76]
	α-Enolase	H H,I H I	PC 3-NT HNE PC	[72] [85] [76] [81]
	Pyruvate kinase	I H	HNE PC	[76] [72]
	Lactate dehydrogenase	H	HNE	[76]
	Glucose-regulated protein precursor	I	3-NT	[85]
	ATP synthase	H,I	HNE	[76]
	Carbonic anhydrase II	I	PC	[79]
Redox Homeostasis & Defense	Peroxiredoxin 6	H	HNE	[76]
	Multidrug resistance protein 3	I	3-NT	[85]
	Glutathione S-transferase Mu	I	3-NT	[85]
	Carbonyl reductase	H	HNE	[76]
Excitotoxicity	Glutamine synthetase	H	PC	[72]
Synaptic plasticity, neurotransmission, cytoskeletal structure	Dihydropyrimidinase-related protein-2 (aka Collapsin response mediated protein-2)	H	3-NT	[79,85]
	Fascin 1	H	3-NT	[85]
	β-Actin	I	HNE	[76]
	Neuropolypeptide h3 (also called Phosphatidyl ethanolamine binding protein 1)	H	HNE	[76]
	Syntaxin-binding protein-1	I	PC	[105]
Protein synthesis	Elongation factor-Tu	I	HNE	[76]
	Initiation factor a	I	HNE	[76]
Protein folding	Heat-shock protein 70	H H I	HNE 3-NT PC	[76] [85] [105]
	Heat-shock protein 90	I	PC	[81]
Signal transduction	Mitogen-activated protein kinase 1	I	PC	[105]
Regulation of protein function	Peptidylprolyl cis-trans isomerase (Pin1)	H	PC	[72]
	14–3–3-γ	I	3-NT	[85]
